# Muscularis macrophages controlled by NLRP3 maintain the homeostasis of excitatory neurons

**DOI:** 10.7150/ijbs.91389

**Published:** 2024-04-15

**Authors:** Yunhuan Gao, Yi Shi, Ming Wei, Xiaorong Yang, Yang Hao, Haifeng Liu, Yuan Zhang, Lu Zhou, Gang Hu, Rongcun Yang

**Affiliations:** 1 Translational Medicine Institute, Tianjin Union Medical Center of Nankai University, Tianjin, 300121, China.; 2 Department of Immunology, Nankai University School of Medicine; Nankai University, Tianjin 300071, China.; 3 State Key Laboratory of Medicinal Chemical Biology, Nankai University, Tianjin 300071, China.; 4 School of Statistics and Data Science, LPMC and KLMDASR, Nankai University, Tianjin, China.; 5 Department of Gastroenterology and Hepatology, General Hospital, Tianjin Medical University, Tianjin, China.

**Keywords:** Muscularis macrophages, NLRP3, excitatory neurons, gut microbiota

## Abstract

Peristaltic movements in gut are essential to propel ingested materials through the gastrointestinal tract. Intestinal resident macrophages play an important role in this physiological function through protecting enteric neurons. However, it is incompletely clear how individuals maintain the homeostasis of gut motility. Here we found that NLRP3 is a critical factor in controlling loss of muscularis resident macrophages (MMs), and demonstrate that MMs are involved in the homeostasis of excitatory neurons such as choline acetyltransferase (ChAT)^+^ and vesicular glutamate transporter 2 (VGLUT2)^+^ but not inhibitory neuronal nitric oxide synthase (nNOS)^+^ neurons. *NLRP3* knockout (KO) mice had enhanced gut motility and increased neurons, especially excitatory ChAT^+^ and VGLUT2^+^ neurons. Single cell analyses showed that there had increased resident macrophages, especially MMs in *NLRP3* KO mice. The MM proportion in the resident macrophages was markedly higher than those in wild-type (WT) or *caspase 1/11* KO mice. Deletion of the MMs and transplantation of the *NLRP3* KO bone marrow cells showed that survival of the gut excitatory ChAT^+^ and VGLUT2^+^ neurons was dependent on the MMs. Gut microbiota metabolites β-hydroxybutyrate (BHB) could promote gut motility through protecting MMs from pyroptosis. Thus, our data suggest that MMs regulated by NLRP3 maintain the homeostasis of excitatory neurons.

## Introduction

Peristaltic movements in gut are essential to propel ingested materials through the gastrointestinal tract. Gastrointestinal dysmotility can induce functional obstruction of the small and large intestine, which is commonly termed as ileus and Ogilvie syndrome, respectively. There are two different networks the submucosa and myenteric plexuses (MPs), which are organized by intrinsic enteric-associated neuron 1. MPs can control muscle movements of gastrointestinal tract. They are composed of multiple heterogeneous neurons such as excitatory acetylcholine transferase (ChAT) and inhibitory neuronal nitric oxide synthase (nNOS) neurons 2, 3. Recent studies have also found that the roles of excitatory vesicular glutamate transporter 2 (VGLUT2)^+^ neurons in the mouse colon, which can be detected in enteric nervous system of mouse colon 4-6.

Intestinal resident macrophages derived from yolk sac macrophages or adult bone-marrow monocytes (BMM) 7-9 play an important role in maintaining normal function of gastrointestinal tract. In gut tissues, BMM-derived macrophages after birth substitute rapidly for yolk-sac-derived intestinal macrophages. However, yolk-sac-derived macrophages also persist and self-renew in the specialized intestinal niches in adults 10, 11. These yolk sac macrophages or adult bone-marrow monocytes derived tissue resident macrophages can express specific makers such as MHCII, F4/80 and CX3CR1, termed CX3CR1 resident macrophages 12-14. Depletion of these gut resident macrophages can result in the abnormalities of the submucosal vasculature and loss of enteric neurons, causing vascular leakage and reduced intestinal motility 11, 15. Gut MPs contain numerous resident macrophages, termed resident muscularis macrophages (MMs), residing in enteric ganglia 15-17. Through direct crosstalk with enteric neurons, these MMs can exert important functions such as effects on the gastrointestinal motility. These MMs can produce bone morphogenetic protein 2 (BMP2), which changes enteric neuron development and influences bowel motility 15, 18, 19. β2-adrenergic receptor (β2-AR) and arginase 1 (arg1) in the MMs are also implicated in enteric neuronal regulation 16, which limits infection-induced neuronal loss 5. Notably, age-dependent effects on residnet macrophages can cause inflammation-mediated degeneration of enteric nervous system 18. These MMs possess different transcriptional profiles, which are demonstrated through single-cell RNA sequencing (scRNA-seq) technologies 20-22. Since MPs are comprised of numerous and heterogeneous neurons, it is unclear whether the MMs have a similar regulation effect on these different neurons.

The homeostasis of macrophages could be controlled by multiple ways such as pyroptosis and necroptosis 23. There are at least five pattern-recognition receptors (PRRs) as sensor proteins to induce pyroptosis in these macrophages, including NLRP3 (NOD-, LRR- and pyrin domain-containing protein 3), NAIP/NLRC4 (NLR family, CARD domain containing4) oligomer, AIM2 (absent in melanoma-2), Pyrin (TRIM20), and NLRP1. These receptors can sense a variety of pathogen-associated molecular patterns (PAMPs), endogenous danger signals and environmental irritants 23, 24. In addition, an alternative pathway to activate pyroptosis is also triggered by caspase-11/4/5, which can be activated by cytosolic lipopolysaccharide (LPS) 25. The MMs, residing in enteric ganglia can be also controlled by these PRRs. But it remains largely unknown which PRR(s) plays a main role in maintaining the homeostasis of the MMs in colon tissues.

NLRP3 inflammasomes as intracellular sensors can sense PAMPs and damage-associated molecular patterns (DAMPs) 26. Both PAMPs and DAMPs can lead to NLRP3 inflammasome assembly, thereby inducing CASPASE-1 mediated inflammatory cytokine maturation and release, and pyroptosis 27. These PAMPs and DAMPs include multiple pathogens such as bacteria, viruses, fungal, protozoan and host-derived moieties such as extracellular ATP, calcium phosphate dihydrate, uric acid crystals, cholesterol crystals, and glucose 26. We here demonstrate that NLRP3 is a main factor in maintaining homeostasis of the MMs in colon tissues. Meanwhile we also found that excitatory ChAT^+^ and VGLUT2^+^ but not inhibitory nNOS^+^ neurons in the MPs can be protected by the MMs.

## Materials and Methods

The reagents and oligoes used in this study are listed in supplementary Table S1.

### Mice

Four-to six-week-old male or female C57BL/6 mice were offered by Nanjing Animal Center. *NLRP3 -/-* and *caspase1/11 -/-* in B6 background were from Prof. Meng in University of Chinese Academy of Sciences, Shanghai, and Prof. Shao in National Institute of Biological Sciences, Beijing. Mice were maintained under specific pathogen-free (SPF) conditions in the Animal Center of Nankai University. Age- and gender- matched mice were used in the experiments, which were approved by the Institute's Animal Ethics Committee of Nankai University (2019-SYDWLL-000600). All procedures were performed according to the Institutional Animal Care and Use Committee of the Model Animal Research Center.

### Mouse models

For deletion of macrophages 28, mice were intraperitoneally (i.p) injected using liposome-encapsulated clodronate (0.1 mL/10 g body weight) three times (Day 1, 3 and 5) a week for 2 weeks. Control mice were injected using equal amounts of PBS-loaded liposomes.

For bone marrow cell (BMC) transplant experiment, recipient CD45.1 mice were irradiated (800 cGy) using a Shepherd Mark I Cesium Irradiator (J.L. Shepherd and Associates). And then CD45.2^+^ BMCs collected from WT, NLRP3 KO or caspase 1/11 KO mice were injected into irradiated CD45.1 recipient mice (5 × 10^6^ cells per mouse) via the tail vein.

For β-hydroxybutyrate (BHB) administration, six-to-eight week-old male mice were treated with BHB-nLGs (i.p 125mg/kg, twice/week), and then mice were analyzed for resident macrophages, neurons and physiology 29. To decrease clearance, BHB was complexed with nanolipogels (nLGs) 30.

### Single-cell RNA-Seq processing

#### Preparation of single cell suspension

Previously reported method 11 was used with modification. Briefly, mice were sacrificed, and colon was removed. Stripped ME was cut and digested in 2 mg/mL collagenase type IV (GIBCO) in RPMI (Sigma) supplemented with 2% HEPES (GIBCO), 2% FBS and DNase (Roche). The remaining pieces containing intestinal LP and submucosa was washed and digested in 0.85 mg/mL collagenase type V (Sigma-Aldrich) in RPMI (Sigma) supplemented with2%HEPES, β-mercaptoethanol (1:1000, GIBCO) and DNase. Percoll gradient separation was performed, and the cells were collected and then stained for sorting and analyzed by flow cytometry. Dead cells were eliminated through 7-AAD staining.

#### Cell capture, cDNA synthesis and single cell RNA-Seq library preparation

Previously method 31 were performed by CapitalBio Technology, Beijing. Briefly, the cell suspension was loaded onto the Chromium single cell controller (10× Genomics) to generate single-cell gel beads. Captured cells were lysed, and RNA were barcoded through reverse transcription and finally the cDNA was generated. Single-cell RNA-seq libraries were prepared using Single Cell 3' Library and Gel Bead Kit V3, and sequenced using an Illumina Novaseq6000 sequencer with a sequencing depth of at least 100,000 reads per cell with pair-end 150 bp (PE150) reading strategy.

### Single-cell RNA-Seq data analyses

#### Single-cell RNA-Seq data preprocessing and clustering with DESC

Single-cell RNA-Seq data preprocessing and clustering with DESC was performed according to previously method 32. Briefly, single-cell RNA-seq data were pre-processed and qualitied with Seurat (version 4) 33. The cells, which had greater than 15% expression originating from mitochondrial genes as well as expressed less than 200 genes were removed. Clustering was analyzed using a Deep Embedding algorithm for Single-cell Clustering (DESC) 34. DESC pretrained an autoencoder and initialized the clustering using Louvain. Then, the software iteratively fine-tuned the encoder and cluster layer to produce final cluster assignment. Two hidden layers with 256 and 32 nodes in the encoder were used, and 2000 highly variable genes were selected using Seurat VST method as the input of DESC. The cell types were found based on the expression of known marker genes. We further clustered resident macrophages with 128 and 64 nodes in the encoder.

### Identification of differentially expressed genes

The differentially expressed genes (DEGs) in each cluster were determined using the Poisson generalized linear model implemented in the Seurat. The genes with log fold-change greater than 0 and Bonferroni-adjusted p-values less than 0.05 were considered DEGs for each cluster and used to subsequent analyses. The enrichment analyses were performed using Metascape (http://metascape.org/) 35.

#### Trajectory analysis with monocle

Monocle2 was used to order all macrophages in pseudotime along a trajectory. Monocle helps discover cells transition from one state to another 36. DEGs for macrophages were determined using the Poisson generalized linear model implemented in the Seurat v4 FindAllMarkers function, and the DEGs with log fold-change larger than 1 and Bonferroni-adjusted p-values less than 0.01 and the top 1000 highly variable genes were used. Then we constructed trajectory and calculated pseudotime following the steps in the monocle2 constructing single cell trajectories tutorial.

#### Plot

Violin plot and feature plot were plotted using VlnPlot function and FeaturePlot function in Seurat V4. DimPlot function in Seurat V4 was used to graph the output of the dimensional reduction on a 2D scatter plot. DoHeatmap function in Seurat V4 was used to draw the heatmap of single cell feature expression.

### Flow cytometry

For flow cytometry, previously reported methods were used 37, 38. Cells were collected, and then incubated with PE-, FITC-, APC- or percy5.5-labeled antibodies for 30 min in PBS with 1% FBS. After washed twice, cells were re-suspended and analyzed using a FACScan flow cytometer. Dead cells were eliminated through 7-AAD staining. Controls were stained using isotopic antibodies.

### Immunofluorescence

Previously reported method by us 37 was used with modification. Briefly, the colon was fixed in 4% (w/v) paraformaldehyde buffered saline, and embedded in paraffin, 5 µm sections colon sections were cut and stained.

Longitudinal muscle strips with the myenteric neuronal plexus from the mice colon were dissected carefully from colonic tissue and fixed in 4% paraformaldehyde as previously published procedures 39. After incubated with blocking buffer, the primary antibodies were added overnight at 4℃, and then secondary Alexa fluor 488- conjugated or Alexa fluor 594-conjugated antibody was added for 1 h at room temperature. The nucleus was stained with DAPI for 3 min. Samples were analyzed using a laser scanning confocal microscope (Leica, Japan).

### Western blot

For western blot, previously reported methods were used 37, 38. Briefly, the cells were harvested, and cell extracts were prepared with lysis buffer. Protein samples were electrophoresed and transferred to PVDF membranes. After the membranes were blocked, and the incubated with first antibody in TBST overnight at 4°C, and then secondary antibodies with horseradish peroxidase (HRP) were added. When HRP substrate was added, the signals were detected by autoradiography film.

### Ex vivo stimulation

For inflammasome activation, the isolated macrophages from the colon tissues of mice were primed with LPS (2μg/mL) for 4 h, and subsequently stimulated with nigericin (5μM) or nigericin with BHB (1mM) for 1 h to activate NLRP3 inflammasomes. Total cell lysates and supernatants were analyzed by immunoblotting.

### Gastrointestinal transit test

Previously reported method 40 was used. Briefly, the mice were fasted for 16 hours before experiment, and then fed with carmine red dye (0.1mL/10 g body weight) next day. After that, the time for expulsion of the first red feces was determined.

### Colonic motility assessment

Previously reported method 34 was used with modification. Briefly, using a plastic Pasteur pipette coated with glycerine enema, a 3-mm glass bead was placed into 2 cm from the anal opening. Distal colonic transit time was detected in fed mice. The mice were fasted for 12 hours before experiment.

### Muscle strip organ bath experiment

Previous reported method 11 was used with modification. Briefly, colonic muscle strip preparations were isolated from mice, and then tested for their longitudinal contractile responses to stimuli. Strip preparations were opened along the mesenteric border and pinned flat in a Sylgard-lined dish. Next, the mucosal layers were removed and strips were cut. Before equilibration at optimal stretch (1.0g), the strips were suspended along their longitudinal axis. Strips were washed at least three times every five minutes. Then ACh (0.05g/ml) was added to the organ bath. Contractions were measured using an isometric force amplifier (Powerlab).

### Quantification of neuron cells

Reported methods were used with modification 11. HuC/D, ChAT, VGLUT2 and nNOS or Cc1 positive neurons were counted in 3 ganglia per myenteric plexus (400× magnification) in the middle colon per animal. A ganglion was defined as a cohesive aggregate of HuC/D+ cells. Extra-ganglionic cells were not counted.

### Statistical analyses

Statistical analyses were performed using two-tailed Student t test, the Mann-Whitney U test and ONE-way ANOVA Bonferroni's Multiple Comparison Test by GraphPad Prism 7 software (GraphPad Software). A 95% confidence interval was considered significant and defined as *, P < 0.05; **, P < 0.01; ***, P < 0.001.

## Results

### NLRP3 KO mice have enhanced gut motility with increased excitatory ChAT^+^ and VGLUT2^+^ neurons in myenteric plexus

**Figure 1:**
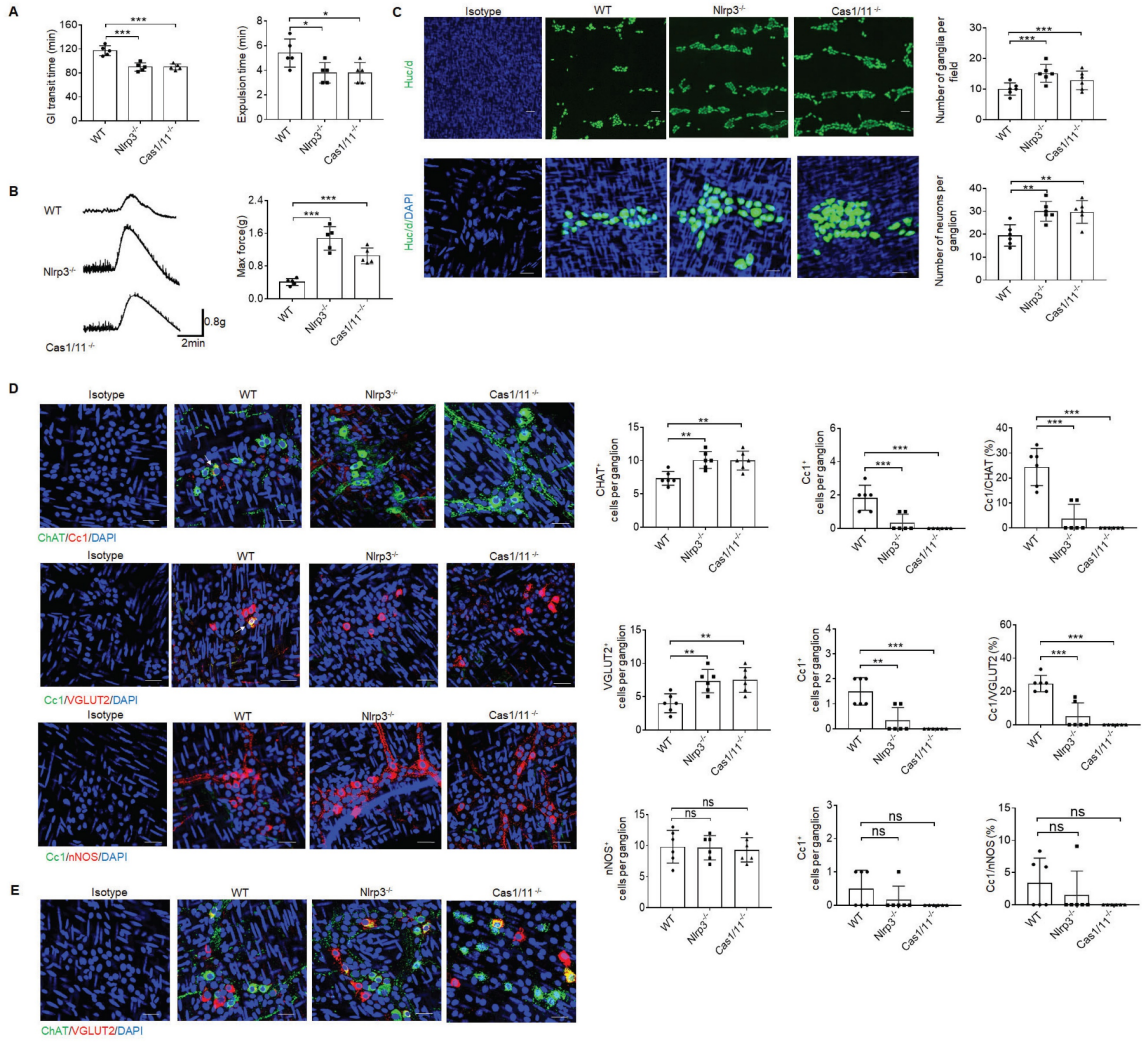
*NLRP3* KO mice have enhanced gut motility with increased ChAT^+^ and VGLUT2^+^ neurons in myenteric plexus. (A) Expulsion analyses of carmine (left) and glass beads (right, n = 5). A representative of at least three experiments. GI, gastrointestinal. (B) Longitudinal contractile responses of colonic muscle strips to stimuli. Colonic muscle strips were isolated from SPF WT, *NLRP3* KO and *CASPASE-1/11* KO mice (n=5). Representative tracings of myogenic contractile responses were induced by ACh (0.05g/ml). A representative of at least three experiments. (C) Immunostaining of HuC/D neurons in MPs of the colon of WT, *NLRP3* KO and *CASPASE-1/11* KO mice. DAPI, blue. Scale bar=25μM. (D) Immunostaining for Cc1/ChAT, Cc1/VGLUT2 and Cc1/nNOS neurons in the ganglia of the MP in the colon of WT, *NLRP3* KO and *CASPASE-1/11* KO mice. € Immunostaining for ChAT and VGLUT2 neurons in the ganglia of the MP in the colon of WT, *NLRP3* KO and *CASPASE-1/11* KO male mice. In C-E, 10 ganglia in the MP per male mouse, n=6 mice. ONE-way ANOVA Bonferroni's Multiple Comparison Test; *P < 0.05, **P < 0.01, ***P < 0.001; Ns, no significance; Nlrp3^-/-^, *NLRP3* KO mice; Cas1/11^-/-^,* CASPASE-1/11* KO mice; Isotype, isotypic antibodies.

Previous studies showed that gut motility could be regulated by CASPASE-11 or NLRP6, which directly affected the pyroptosis of neurons 5, 34, 41. To investigate the effect(s) of another inflammasome NLRP3 on gut physiological motility, we performed gastrointestinal transit test using carmine red dye and glass beads. The time of eliminating carmine or glass beads was significantly shorter in *NLRP3* KO mice than control wild-type (WT) mice (Figure 1A), indicating that GI motility is upregulated. Indeed, enhanced gut force upon exposure to muscarinic agonist acetylcholine (ACh) was observed in *NLRP3* KO mice (Figure 1B), which was similar to control* CASPASE-1/11* KO mice 41. Gut motility is related to the numbers of enteric neurons 5, 41. There are two distinct networks, the submucosal and MPs in the enteric nervous system 1. More myenteric neurons could be detected in *NLRP3* KO mice (Figure 1C). MPs are comprised of multiple heterogeneous neurons 1 such as excitatory ChAT^+^ and VGLUT2^+^ and inhibitory nNOS^+^ neurons 5, 11, 42. Data showed that more excitatory ChAT^+^ and VGLUT2^+^ neurons were located in the muscularis externa (ME) of *NLRP3* KO mice as compared to WT mice (Figure 1D). Notably, only some excitatory neurons could co-express ChAT and VGLUT2 (Figure 1E), which was distinguished from previous reports that VGLUT2^+^ neurons are ChAT positive 43. We next used anti-cleaved CASPASE-1 (Cc1) antibody and Gasdermin D (GSDMD) NT to detect pyroptotic neurons 44. Pyroptosis could be found in ChAT^+^ and VGLUT2^+^ neurons of the MPs in WT but not *NLRP3* KO mice (Figure 1D and supplementary Fig. S1), implying that there indeed had increased ChAT^+^ and VGLUT2^+^ neurons in the MPs of *NLRP3* KO mice. These results suggest that there might exist regulation effects of NLRP3 on neurons. Notably, inhibitory nNOS^+^ neurons, which play an important role in smooth muscle relaxation 45, revealed no significant changes in these mice (Figure 1D). As a positive control, *CASPASE-1/11* KO mice also had increased neurons in the colon tissues (Figure 1D). Taken together, *NLRP3* KO mice have enhanced gut motility with increased excitatory ChAT^+^ and VGLUT2^+^ neurons in the MPs.

### NLRP3 KO mice have increased resident macrophages in colon tissues

**Figure 2:**
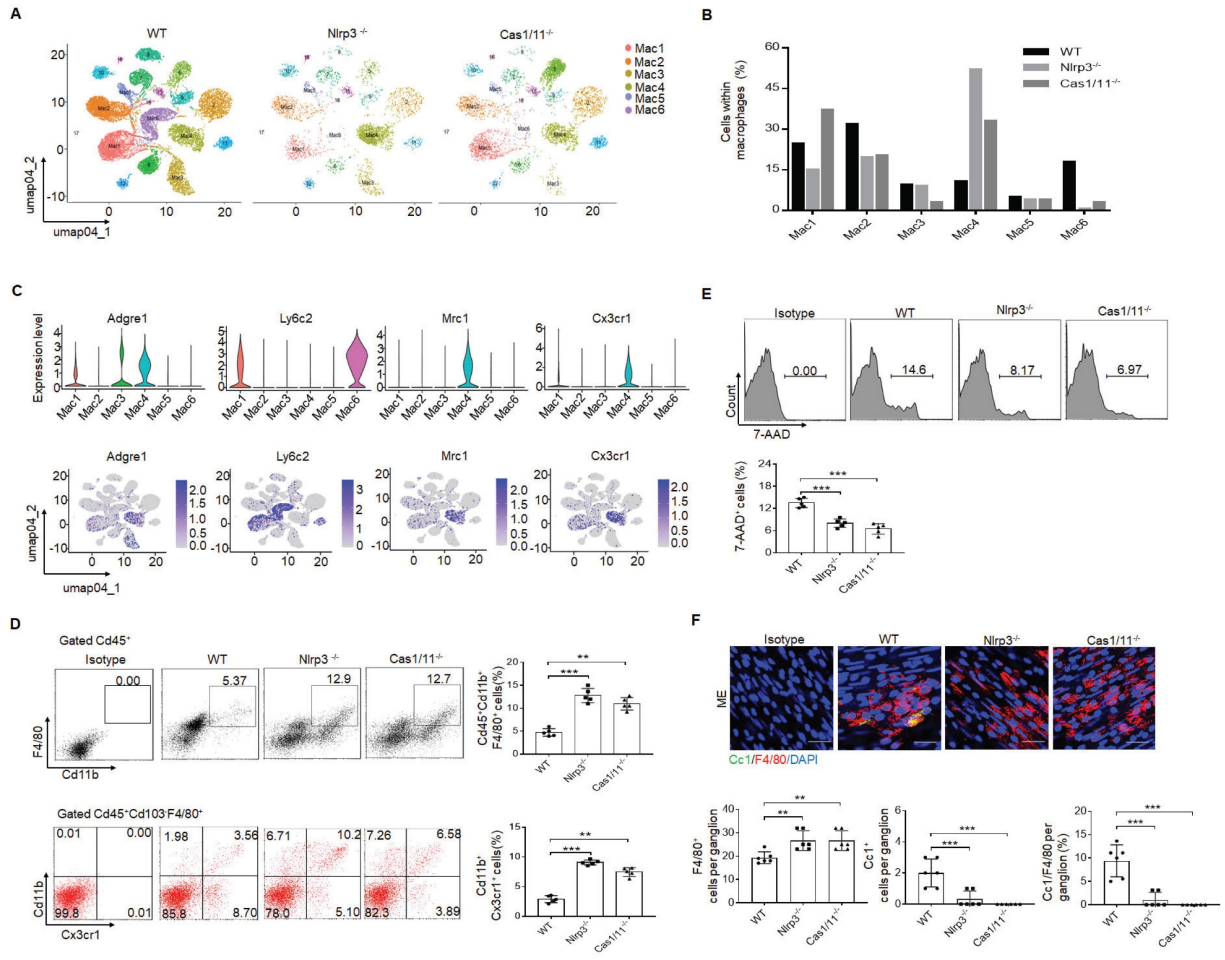
Enhanced gut motility in *NLRP3* KO mice is related to increased resident macrophages. (A) DESC clustering of CD11b^+^ single cells in the colon tissues of control SPF WT mice (n=19895), *NLRP3* KO mice (n=3245) and *CASPASE-1/11* KO mice (n=4294), which were partitioned into 19 distinct clusters. Pooled sample from eight weeks-old male mice, n=6. (B) Proportions of the populations of different macrophages in total CD11b^+^ cells from the colon tissues of control SPF WT, *NLRP3* KO and *CASPASE-1/11* KO mice. (C) Violin plots (upper) and feature plots (lower) showing expression levels of canonical marker genes in the macrophages across different clusters. (D) Flow cytometry of CD45^+^CD11b^+^F4/80^+^ and CD45^+^CD103^-^F4/80^+^ CX3CR1^+^CD11b^+^ cells in the colon tissues of control SPF WT,* NLRP3* KO and *CASPASE-1/11* KO mice (n=5). (E) Flow cytometry of 7-AAD^+^cells in the macrophages of colon tissues of SPF WT, *NLRP3* KO and *CASPASE-1/11* KO mice (n=5). (F) Immunostaining of Cc1 and F4/80 in the MP of the ME in the middle colon in SPF WT, *NLRP3* KO and *CASPASE-1/11* KO mice. 10 ganglia in the MP per mouse, n=6; DAPI, blue; Scale bar=25μM; ONE-way ANOVA Bonferroni's Multiple Comparison Test; *P < 0.05, **P < 0.01, ***P < 0.001; Nlrp3^-/-^, *NLRP3* KO mice; Cas1/11^-/-^,* CASPASE-1/11* KO mice; Isotype, isotypic antibodies.

Since gut neurons are regulated by CASPASE-11 or NLRP6 through pyroptosis 5, 34, it is also possible for these neurons to be regulated by another inflammasome NLRP3. However, NLRP3 was not detected in the MP neurons of the mouse colon tissues, indicating that pyroptosis of gut neurons is not regulated by NLRP3 (Supplementary Fig. S2). Intestinal resident macrophage populations play a role in the normal function of enteric neurons 5, 11, 15. The MMs, located within and surrounding the MP, were shown to regulate the activity of enteric neurons and peristalsis 15, 46. Furthermore, the number of neurons was related to the resident macrophages, which could protect neurons 5, 15. Thus, we analyzed these resident macrophages. CD45^+^CD11b^+^ cells were first sorted from the colonic tissues, including submucosa, lamina propria (LP) and muscularis externa (ME) of specific pathogen free (SPF) WT, *NLRP3* KO and also *CASPASE-1/11* KO mice (6-8 weeks old, male mice). Then sorted CD45^+^CD11b^+^ cells from pooled sample (6 mice) were sequenced on a 10× Genomics platform (a single cell RNA sequencing (scRNA-seq) platform) and clustered using DESC, a deep embedding algorithm for single-cell clustering 34. The macrophages in the colon tissues includes 6 subpopulations (Figure 2A), similar to other analyses 34. Importantly, the cluster 4 subpopulation was markedly increased in the colon tissues of *NLRP3* KO mice (Figure 2B), which also cause reduced proportion of other subpopulation in mice. This population of macrophages expressed high levels of F4/80^+^, Mrc1 (CD206^+^) and CX3CR1^+^ (Figure 2C), which can be detected by resident macrophages, indicating that increased cluster 4 macrophages in *NLRP3* KO mice are CX3CR1^+^resident macrophages 12-14. Cluster 6 and 1 expressed high levels of Ly6c2 and F10 (Figure 2C and supplementary Fig. S3), which were the markers of blood monocytes/macrophages 47, suggesting that these clusters belong to monocytes/macrophages. Previous studies indicated that Ly6C^hi^ monocytes were able to enter into the colon, and then mature into F4/80^hi^CX3CR1^hi^MHCII^+^ CD64^+^ resident macrophages 48. This developmental process involves a series of identifiable intermediaries in which CCR2, F10, Ly6c2, Hdc, Sell and Hp, markers of blood monocytes 47 are lost, while expressions of F4/80, CX3CR1, CD206, CD163 and CD64 are gained or upregulated 9, 49. Our results supported this progression of monocyte differentiation through distinct phenotypic developmental stages (Supplementary Fig. S3). However, yolk sac derived macrophages also express CX3CR1 50. Thus, CX3CR1^+^resident macrophages in gut tissues identified by scRNA-seq might come from both bone marrow monocytes or yolk sac. Increased CX3CR1 resident macrophages in the colon tissues of *NLRP3* KO mice were further confirmed using flow cytometry and immune staining (Figure 2D and supplementary Fig. S4). These increased resident macrophages were related to reduced macrophage pyropotosis (Figure 2E, F). Indeed, *NLRP3* KO resident macrophages from the colon tissues were more resistance to NLRP3 mediated pyroptosis (LPS plus nigericin) (Supplementary Fig. S5). In addition, F4/80^+^ macrophages could be detected in whole colon tissues; Whereas Ly6C macrophages did not be detected in the muscularis externa (ME) (Supplementary Fig. S6), suggesting that Ly6C^+^ macrophages, which belong to inflammatory macrophages 9, 49, do not contribute to the ME. Taken together, our data demonstrate that there are increased CX3CR1^+^ resident macrophages in the colon tissues of *NLRP3* KO mice.

**Figure 3:**
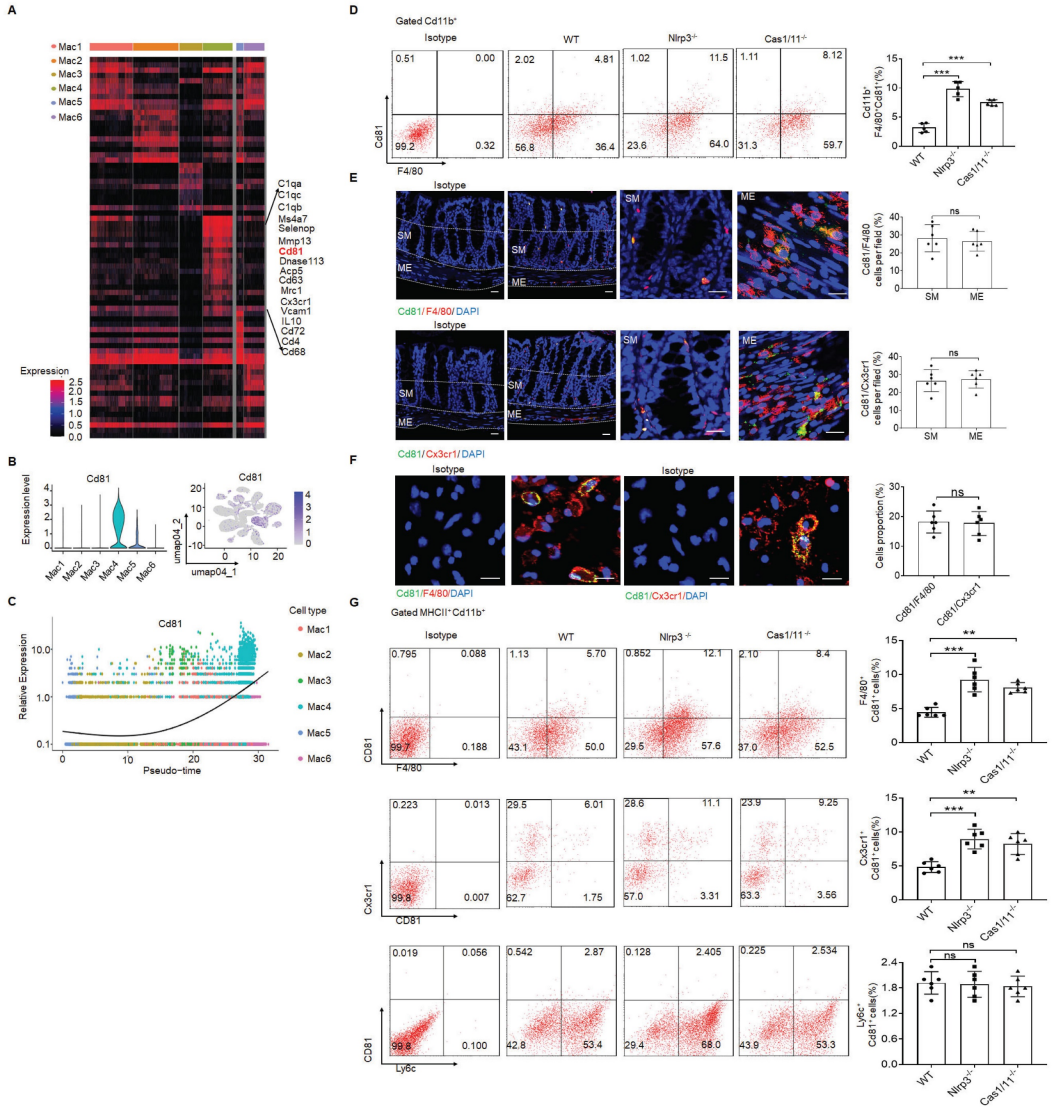
CD81 is a surface marker of gut resident macrophages. (A) Heatmap reporting differentially expressed genes of different macrophage populations. The differential expression analysis is performed using Poisson generalized linear model implemented in Seurat. (B) Violin plots (left) and feature plots (right) showing gene expression level of CD81. (C) Monocle 2 trajectory analysis of CD81 from different macrophage subsets during their development. (D) Flow-cytometry of CD45^+^ CD11b^+^ F4/80^+^CD81^+^ cells in the isolated cells from the colon tissues of SPF WT, *NLRP3* KO and *CASPASE-1/11* KO mice (n=5). € Immunostaining of F4/80, Cx3cr1 and CD81 in submucosa and ME of whole mount colon sections from mice. 3 fields (100×) per mouse; n=6 mice. Scale bar=100 μM (SM+ME), 40 μM (SM) and 25 μM (ME). Dapi, blue. (F) Immunostaining for F4/80, CD81 and CX3CR1 in isolated CD11b^+^ cells by MicroBeads from the colon tissues of mice. n=6, scale bar= 25 μM. (G) Flow cytometry of F4/80^+^CD81^+^, CD81^+^CX3CR1^+^ and Ly6c^+^CD81^+^ cells in the isolated MHCII^+^ and CD11b^+^ immune cells from the colon tissues of mice (n=6). ONE-way ANOVA Bonferroni's Multiple Comparison Test in D and G; Mann-Whitney U test in E; Student's t-test, mean ±SD in F; *P < 0.05, **P < 0.01, ***P < 0.001; Nlrp3^-/-^, *NLRP3* KO mice; Cas1/11^-/-^,* CASPASE-1/11* KO mice; Isotype, isotypic antibodies.

In addition, *CD81* gene could be detected in the resident macrophage cluster (Figure 3A-C). CD81, as a member of the tetraspanin family, encompasses membrane proteins with four transmembrane domains 51. CD81 marker in the resident macrophages could also be confirmed by flow cytometry and immunostaining (Figure 3D-F), suggesting that CD81 is a surface marker of gut resident macrophages, consistent with the resident macrophages in kidney 52. Notably, only partly F4/80^+^ or CX3CR1^+^ resident macrophages could express surface CD81 (Figure 3G).

### Increased resident macrophages in the colon tissues of NLRP3 KO mice are muscularis macrophages

**Figure 4:**
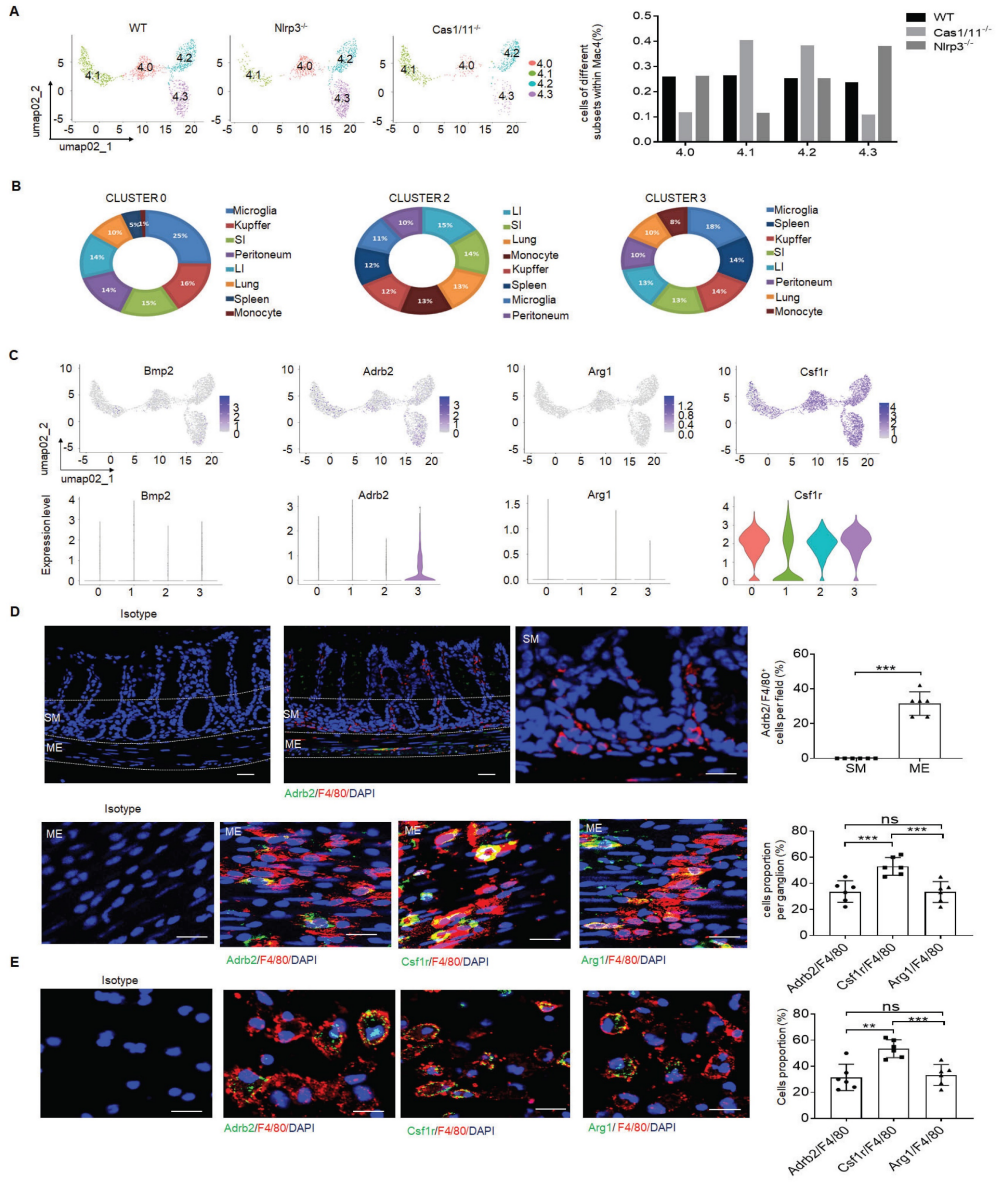
*NLRP3* KO mice have markedly increased MMs. (A) DESC clustering of resident macrophages cluster 4 (left), which were partitioned into 4 distinct clusters (cluster 4.0, 4.1, 4.2 and 4.3). Proportion of different subsets in resident macrophages cluster 4 of SPF WT,* NLRP3* KO and *CASPASE-1/11* KO mice (right). (B) The average expression proportion of marker genes identified in cluster 0 (4.0), 2 (4.2) and 3 (4.3) of cell type in the ImmGen dataset. Distribution of marker genes identified in cluster in cluster 0 (4.0), 2 (4.2) and 3 (4.3) across different tissue-resident macrophages, as identified by the ImmGen dataset. (C) Feature plots (upper) and violin plots (lower) showing gene expression level of marker genes in different subpopulations (0, 4.0; 1,4.1; 2, 4.2;3, 4.3) of resident macrophages cluster 4. (D) Immunostaining of F4/80 with adrb2, arg1 and csf1r in submucosa (SM) or muscularis externa (ME) of the colon sections from mice. 10 ganglia in the MP per mouse; n=6 mice. Scale bar=100μm (SM+ME), 40μm (SM) and 25μm (ME). DAPI, blue. (E) Immunostaining of F4/80 with adrb2, csf1r and arg1 in the isolated CD11b^+^cells from the colon tissues of SPF WT mice by MicroBeads (n=6). Scale bar= 25 μm. Mann-Whitney U test in D; ONE-way ANOVA Bonferroni's Multiple Comparison Test in E; *P < 0.05, **P < 0.01, ***P < 0.001; Nlrp3^-/-^, *NLRP3* KO mice; Cas1/11^-/-^,* CASPASE-1/11* KO mice; Isotype, isotypic antibodies.

There existed multiple resident macrophage populations such as mucosal macrophages, perivascular macrophages, crypt base macrophages and MMs in the colon tissues of mice 16. Indeed, the resident macrophages in the cluster 4 of Figure 2 could further be divided into 4 subsets by DESC, including cluster 4.0, 4.1, 4.2 and 4.3 (Figure 4A and supplementary Fig. S7A). Interestingly, while the proportion of four subpopulations of resident macrophages in WT mice was basically equal, cluster 4.3 subpopulation in *NLRP3* KO mice was markedly increased (Figure 4A). This was also different from control *CASPASE-1/11* KO mice, which had markedly increased resident macrophages in cluster 4.1 and 4.2, but much less resident macrophages in cluster 4.3 (Figure 4A). Since activation of CASPASE-11 or NLRP3 inflammasomes upon exposure to their ligands can cause the loss of the macrophages 24, we speculate that increased cluster 4.1 and 4.2 in *CASPASE-1/11* KO mice might be located in mucosa or submucosa since there have more LPS 53, which can cause pyroptosis of macrophages; Whereas increased cluster 4.3 macrophages in* NLRP3* KO mice might be in the places far from gut cavity such as ME.

We further analyzed these subpopulations based on the marker genes identified in different clusters of cell type using a published RNA-seq database ImmGen. Of note, cluster 4.0 and 4.3 respectively cover 25% or 18% genes with microglia (Figure 4B) 16, 54, implying that these subpopulations potentially are related to neurons. Bio-informatics analyses also confirmed that there indeed existed a signal pathway for the regulation of neuron death in cluster 4.3 (Supplementary Fig. S7B). The resident macrophages of cluster 4.3 but not others could express high levels of β2-AR (adrb2) (Figure 4C, D), suggesting that this subpopulation belongs to MMs 5, 16. Furthermore, arg-1, which could be expressed in MMs, was also detected in these MMs (Figure 4D, E). The development of resident macrophages is dependent on colony stimulatory factor 1 receptor (CSF1R) 15. CSF1R could be also detected in the macrophages surrounding the MP (Figure 4D). However, BMP2, which could stimulate the neurons and influence gut motility 15, 55, was not highly expressed in this subpopulation (Figure 4C). Consistent with other reports 5, 11, 16, resident MM cluster 4 not only expressed F4/80, CX3CR1, CD206, CD163 but also c1qa, c1qb and c1qc (Supplementary Fig. S8A-C) 52, 56. Cluster 4.3 could also co-express *F4/80, CX3XR1, CD206, CD81* and* c1qa, c1qb and c1qc*. Immunostaining showed that c1qa^+^F4/80^+^ macrophages not only existed in mucosa and submucosa but also ME of colon tissues (Supplementary Fig. S8D-F). Furthermore, c1qa^+^ macrophages in the ME could also express β2-AR (adrb2) and CSF1R (Supplementary Fig. S8D-F). Taken together, increased resident macrophages of cluster 4.3 in *NLRP3* KO mice belong to the MMs, which express not only F4/80, β2-AR, arg-1 and CSF1R but also c1qa^+^.

### Effects of NLRP3 on excitatory neurons depend on muscularis resident macrophages

**Figure 5:**
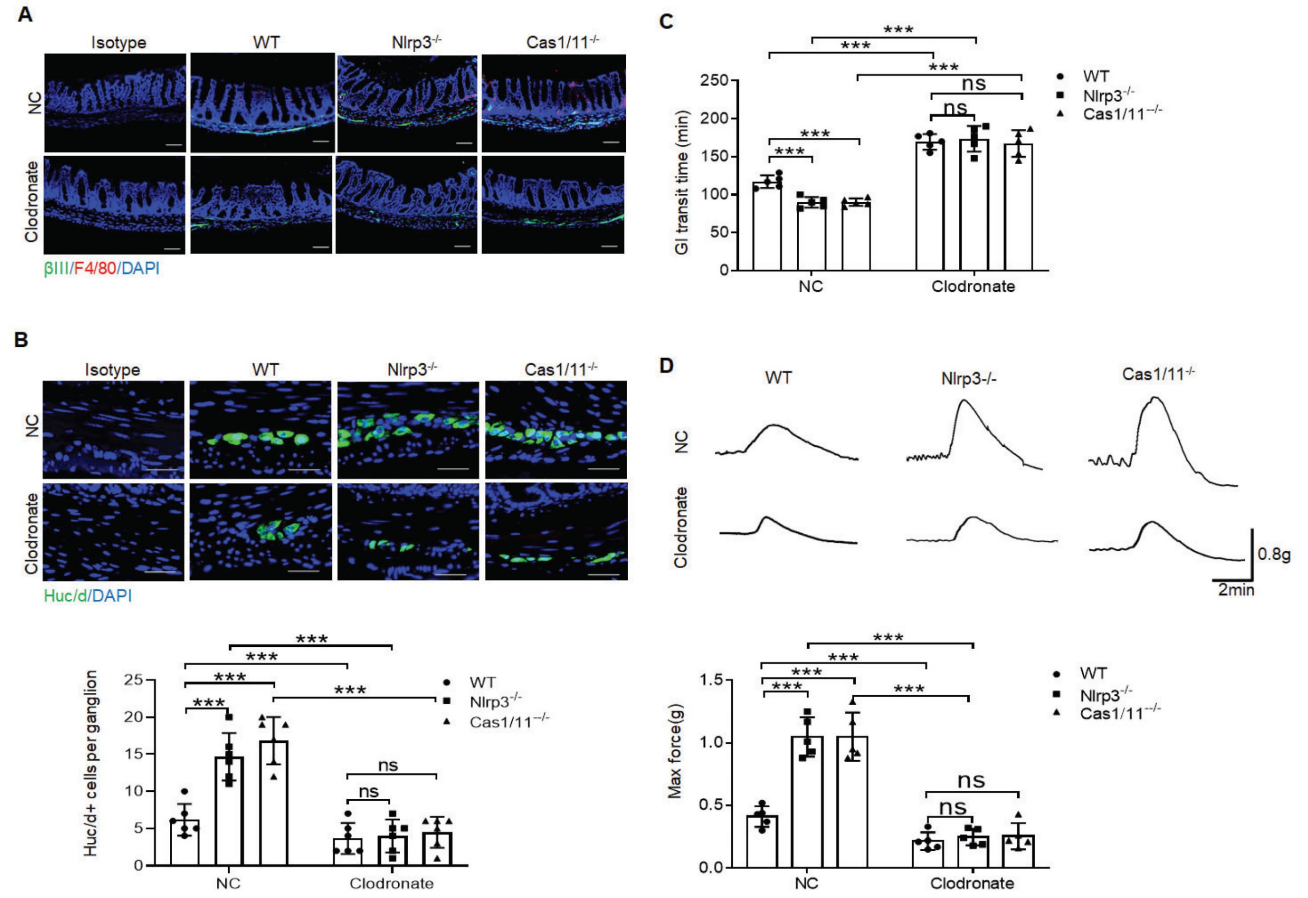
Gut motility depends on the MMs. (A) Immunostaining for F4/80, βIII-Tub and DAPI in the colon sections of clodronate treated (Clodronate) or untreated (NC) control SPF, *NLRP3* KO and *CASPASE-1/11* KO mice. One representative of 6 mice. (B) Immunostaining of HuC/D^+^ cells in the ME of colon sections from clodronate treated (Clodronate) or untreated (NC) control SPF, *NLRP3* KO and *CASPASE-1/11* KO mice. 10 ganglia in the MP per mouse, n=6 mice. Scale bar= 25μm. (C) Total gastrointestinal transit time to expel feces containing carmine (n=5). A representative of at least three experiments. (D) Colonic muscle strip preparations isolated from clodronate treated (Clodronate) or untreated (NC) mice. Representative tracings of myogenic contractile responses were induced by ACh (0.05g/ml) in clodronate treated (Clodronate) or untreated (NC) control SPF, *NLRP3* KO and *CASPASE-1/11* KO mice. One representative of at least three experiments. ONE-way ANOVA Bonferroni's Multiple Comparison Test; *P < 0.05, **P < 0.01, ***P < 0.001; Nlrp3^-/-^, *NLRP3* KO mice; Cas1/11^-/-^,* CASPASE-1/11* KO mice.

Depletion of MMs results in loss of enteric neurons 11. To further determine protective roles of the resident macrophages in the neurons, we deleted macrophages using liposome encapsulated dichloromethylene with demonstrated deletion of macrophages (Figure 5A). Consistent with previous reporter 15 at 2 days after deleting macrophages, there are no significant changes in HuC/D^+^ enteric neurons. However, over 7 days after deletion of macrophages, there had markedly reduced HuC/D^+^ enteric neurons in MP (Figure 5B), indicating that resident macrophages determine the loss of enteric neurons in MPs. Deletion of macrophages in mice caused also decreased elimination time of carmine and maximal contractile forces in response to ACh (Figure 5C, D). Thus, resident macrophages were necessary for excitatory enteric neurons.

**Figure 6:**
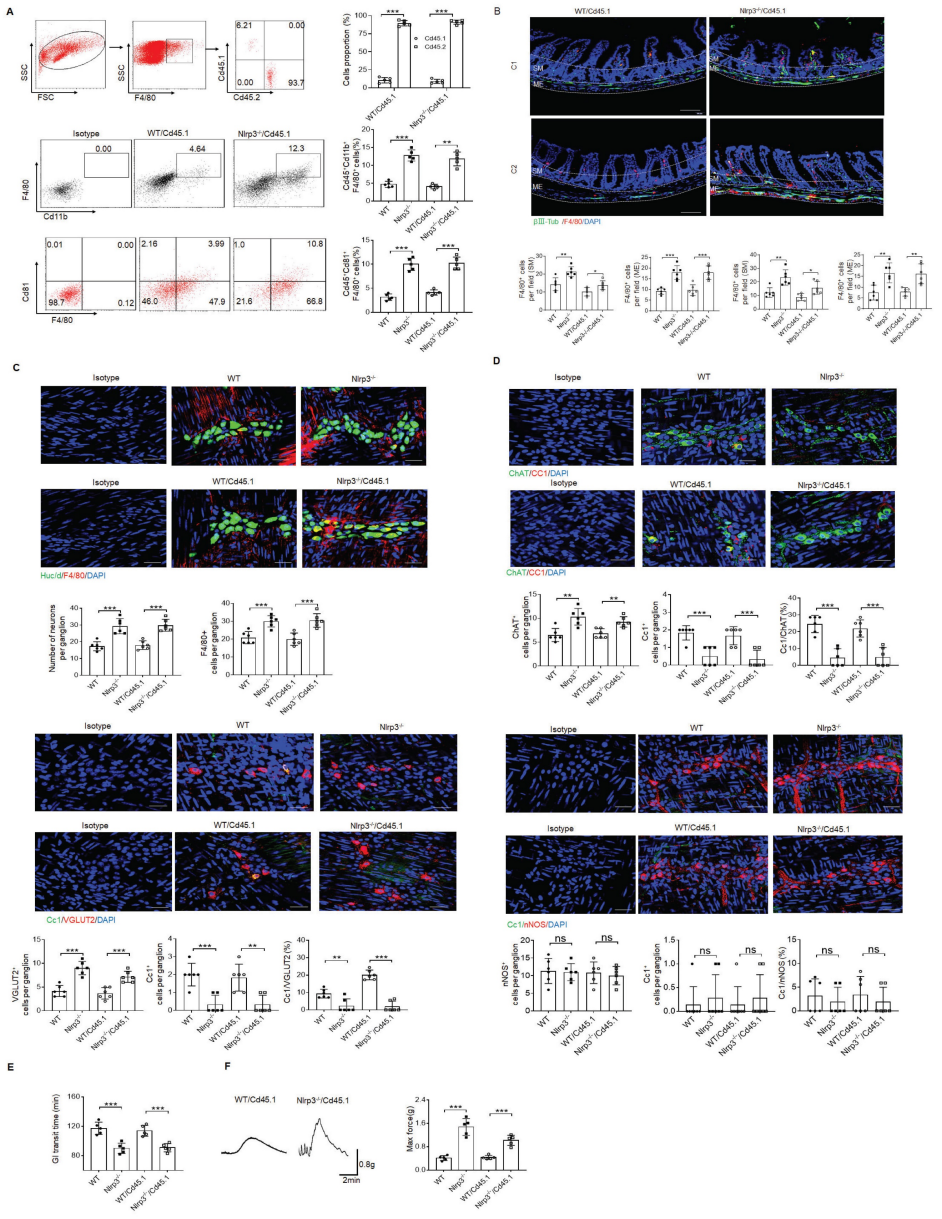
Enhanced motility in *NLRP3* KO mice depends on the MMs. (A) Flow cytometry of CD11b^+^F4/80^+^ and F4/80^+^/CD81^+^ in isolated cells from the colon tissues of chimeric mouse models of WT/CD45.1 and NLRP3 KO/CD45.1 mice (n=5). (B) Immunostaining for F4/80, βIII-Tub and DAPI in the colon sections from WT/CD45.1 and NLRP3(Nlrp3) KO/CD45.1 mice. C1, near cecum; C2, near anus. Scale bar=100 μm; 3 fields (100×) per mouse; n=6 mice. (C) Immunostaining of HuC/D^+^ and F4/80^+^cells in the MP from WT /CD45.1 and NLRP3KO /CD45.1 mice. 10 ganglia in the myenteric plexus per mouse, n=6 mice. Scale bar= 25μm. (D) Immunostaining of Cc1/ChAT, Cc1/VGLUT2 and Cc1/nNOS neurons in the MP of the colon of WT/CD45.1 and NLRP3 KO /CD45.1 mice. 10 ganglia in the MP per mouse, n=6 mice. Scale bar= 25μm. (E) Gastrointestinal transit time to expel feces containing carmine (n=5). A representative of at least three experiments. (F) Longitudinal contractile responses of colonic muscle strips to stimuli. Colonic muscle strips isolated from WT/CD45.1 and NLRP3 KO /CD45.1 mice (n=5). Representative tracings of myogenic contractile responses were induced by ACh (0.05g/ml). A representative of at least three experiments. CD45.1 mice were first irradiated and then infused by CD45.2 BMCs of *NLRP3* KO or control SPF WT mice. After 4 weeks, WT /CD45.1 and NLRP3 KO /CD45.1 chimeric mice were prepared. ONE-way ANOVA Bonferroni's Multiple Comparison Test; *P < 0.05, **P < 0.01, ***P < 0.001; Isotype, isotypic control.

MMs can be efficiently repopulated by bone marrow monocytes (BMC) 11. To further illustrate the role of *NLRP3* KO resident macrophages in ChAT^+^ and VGLUT2^+^ neurons in the MPs, we employed BMC transplant model. After transplantation from CD45.2^+^ *NLRP3* KO to CD45.1^+^ mice for four weeks, resident macrophages in the colon tissues of CD45.1 mice were CD45.2^+^ cells, indicating that CD45.1 resident macrophages are replaced by *NLRP3* KO CD45.2^+^ macrophages (Figure 6A). *NLRP3* KO macrophages might resist against NLRP3 ligands surrounding MPs 26. Indeed, there had increased MMs in the mice transplanted by *NLRC3* KO CD45.2^+^ BMCs (Figure 6B). The markedly increased neurons, especially excitatory ChAT^+^ and VGLUT2^+^ neurons around the MPs in the mice transplanted by *NLRP3* KO CD45.2 BMCs were also observed (Figure 6C, D and supplementary Fig. S9). However, the quantification of nNOS^+^ neurons did not reveal significantly changes in the mice transplanted by *NLRP3* KO or WT CD45.2 BMCs (Figure 6D). The increased *NLRP3* KO CD45.2^+^ resident macrophages could also promote not only the elimination of carmine but also enhance force developed by isolated colon muscle strips in response to ACh (Figure 6E, F). Taken together, the effects of NLRP3 on ChAT^+^ and VGLUT2^+^ neurons in the MPs depend on gut MMs.

### More excitatory neurons in the colon tissues of mice treated by gut microbiota metabolite β-hydroxybutyrate

**Figure 7:**
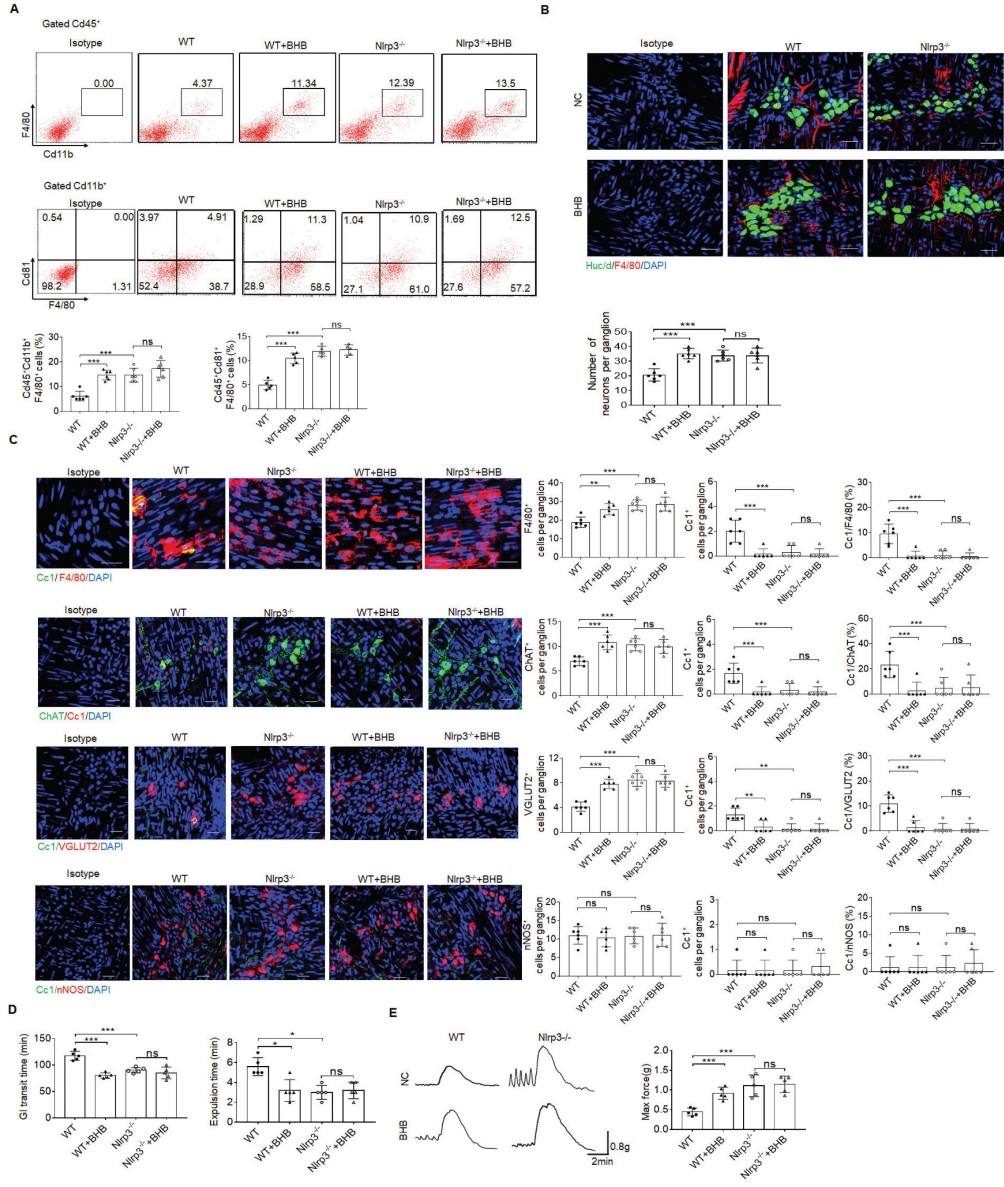
More excitatory neurons in the colon tissues of the mice treated by gut microbiota metabolite β-hydroxybutyrate. (A) Flow cytometry of CD11b^+^F4/80^+^ and F4/80^+^CD81^+^ cells in isolated cells of the colon tissues from β-hydroxybutyrate (BHB) treated *NLRP3* KO and SPF WT mice and control mice (n=5). (B) Immunostaining of F4/80 and HuC/D in the MP of colon tissues from BHB treated *NLRP3* KO and WT mice. 10 ganglia in the MP per mouse, n=6 mice. Scale bar=25 μM. DAPI, blue. (C) Immunostaining of Cc1/F4/80, Cc1/ChAT, Cc1/VGLUT2 and Cc1/nNOS neurons in the MP of BHB treated *NLRP3* KO and WT mice. 10 ganglia in the MP per mouse, n=6 mice. Scale bar=25 μM. DAPI, blue. (D) The expulsion analyses of carmine (left) and glass beads (right) in BHB treated mice and control mice (n=5). A representative of at least three experiments. (E) Longitudinal contractile responses of colonic muscle strips to stimuli. Colonic muscle strips isolated from BHB treated mice and control mice (n=5). Representative tracings of myogenic contractile responses were induced by ACh (0.05g/ml). A representative of at least three experiments. ONE-way ANOVA Bonferroni's Multiple Comparison Test; *P < 0.05, **P < 0.01, ***P < 0.001; Isotype, isotypic antibodies.

Age-related changes in gut microbiota can change the phenotype of MMs and disrupt gastrointestinal motility 57. Thus, it is possible for gut microbiota to regulate these MMs. Gut microbiota metabolite β-hydroxybutyrate (BHB) can suppress activation of NLRP3 inflammasomes in response to urate crystals, ATP and lipotoxic fatty acids 29. Since NLRP3 may exert a critical role in controlling the loss of MMs, we observed whether gut microbiota BHB could resist the loss of MMs to maintain the homeostasis of ChAT^+^ and VGLUT2^+^ neurons. Indeed, flow cytometry and immune-staining showed that F4/80^+^CX3CR1^+^and F4/80^+^CD81^+^resident macrophages, and MMs markedly increased in the colon tissues of WT but not *NLRP3* KO mice after BHB (Figure 7A, B). Excitatory ChAT^+^ and VGLUT2^+^ but not inhibitory nNOS^+^ neurons significantly increased in WT but not *NLRP3* KO mice after administrating BHB (Figure 7B, C and supplementary Fig. S10). Notably, CC1-expressing neurons per ganglia in the WT show significant variation, from an average of ~1.6 cells per ganglia (in ChAT experiment) to 1 cell per ganglia (in VGLUT experiment) to 0.3 cells per ganglia (in NOS1 experiment), implying different responses of different neurons to macrophage' effects. BHB also promoted elimination of carmine and glass beads, and enhanced force developed by isolated colon muscle strips in response to ACh in WT but not *NLRP3* KO mice (Figure 7D, E). We also examined the *in vitro* impact of BHB on the resident macrophages, which were isolated from the colon tissues of mice. Consistent with previous results 29, BHB could markedly decrease the pyroptosis of these resident macrophages through inhibiting nigericin-mediated NLRP3 activation (Supplementary Fig. S11). Taken together, BHB can increase ChAT^+^ and VGLUT2^+^ neurons in the MP of colon tissues through inhibiting pyropotosis of the MMs.

## Discussion

We here found that the MMs can control the loss of ChAT^+^ and VGLUT2^+^ neurons to maintain the homeostasis of gut motility. The MMs are characterized by their tissue-protective signatures such as the expression of *Arg1*, *Ald1a2, Cd163*, *Ccl17*, and *Retnla* 5, 11, 16. MMs identified by us can also express similar markers, belonging to the same kind of resident macrophage subset. These MMs play a critical role in the normal function of enteric neurons. They can produce BMP2 15, β2-AR and Arg1 16 to limits neuronal loss 5. Enteric neurons include multiple kinds of neurons such as excitatory and inhibitory neurons, but it is unclear whether these MMs have a similar role in these neurons. We here demonstrate that the MMs have a regulating role in the excitatory ChAT^+^ and VGLUT2^+^ but not inhibitory nNOS^+^ neurons.

Inhibitory nNOS^+^ neurons might be protected by nitric oxide (NO) produced by themselves. Indeed, previous studies found that NO had neuroprotective properties in nNOS^+^ neurons 58, 59. Others also found that nNOS expression can protect the enteric nervous system 60. Reduced expression of nNOS also leads to the loss of nitrergic neurons 61. NO predominantly synthesized by endothelial nitric oxide synthase (eNOS) in endothelial cells also had an anti‐apoptotic effect 62. Notably, high concentrations of NO in pathophysiological conditions also exhibit pro-apoptotic effects 63. The anti-apoptotic effects are mainly mediated by low amounts of NO 63.

We also demonstrate that NLRP3 plays a critical role in maintaining homeostasis of the MMs. Increased MMs can promote the survival of the neurons in myenteric plexuses 5, 15. The markedly increased MMs, which can increase the homeostasis of excitatory neurons, can be found in the colon tissues of *NLRP3* KO mice. The inflammasomes such as CASPASE-11 and NLRP3, which are expressed in the macrophages, may induce pyroptosis of the macrophages upon exposure to their ligands. There have more LPS in mucosa and submucosa closed to gut cavity 53, in which activated CASPASE-11 can result in the pyroptosis of more resident macrophages. Indeed, there have more macrophages in the mucosa and submucosa of *CASPASE-1/11* KO mice. Whereas NLRP3, which can detect a broad range of microbial motifs, endogenous danger signals and environmental irritants 24, might play a main role in maintaining homeostasis of the MMs in the ME, which is far from gut cavity.

Intestinal peristalsis is a dynamic physiologic process influenced by dietary and microbial changes 15. Microbial-derived metabolites such as short chain fatty acids and tryptophan and bile acid metabolites, interact with various metabolite receptors expressed on enterocytes or enteroendocrine cells (EEC) to regulate gut homeostasis 64. In addition, microbial-derived metabolites have widely effects on the immune cells such as macrophages 65, 66. Since neuron-macrophage interaction disorder may affect the homeostasis of the enteric nervous system (ENS), thereby leading to gastrointestinal dysfunction 67, microbial-derived metabolites also affect the gut motility. Age-related changes in gut microbiota alter phenotype of the MMs and disrupt gastrointestinal motility 57. However, the underlying mechanisms involved in this process is largely lacking 5. We demonstrate that gut microbiota metabolite BHB can regulate gut motility through limiting NLRP3-mediated pyroptosis of MMs. This might have an implication in understanding how gut microbiota alters phenotype of the MMs and disrupts gastrointestinal motility.

## Supplementary Material

Supplementary figures and table.
