# Supplementary material for: Muscularis macrophages controlled by NLRP3 maintain the homeostasis of excitatory neurons
Source: Int J Biol Sci. 2024 Apr 15;20(7):2476–90. doi: 10.7150/ijbs.91389 (PMC11077366; doi:10.7150/ijbs.91389)
Supplement: Supplementary file 1 — Supplementary figures and table. [file ijbsv20p2476s1.pdf]

Supplementary materials

Muscularis macrophages controlled by NLRP3 maintain the homeostasis of excitatory neurons

Yunhuan Gao[1,2,3*], Yi Shi[4*], Ming Wei[1,2,3], Xiaorong Yang[1,2,3], Yang Hao[1,2,3], Haifeng Liu[5], Yuan Zhang[1,2,3], Lu Zhou[5], Gang Hu[4], and Rongcun Yang[1,2,3]

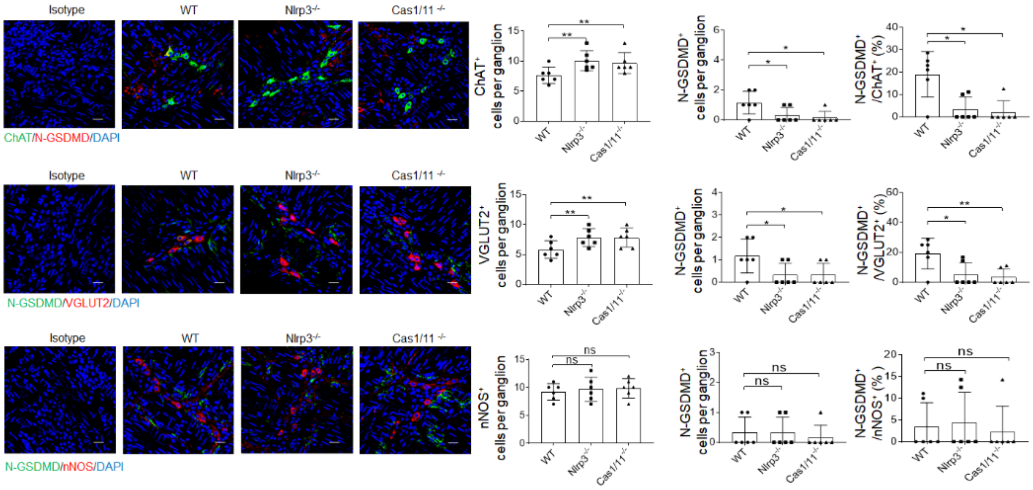

Figure S1. NLRP3 KO mice have increased ChAT+ and VGLUT2+ neurons in myenteric plexus. N-GSDMD immunostaining for ChAT (N-GSDMD/ChAT), VGLUT2 (N-GSDMD/VGLUT2) and nNOS (N-GSDMD/nNOS) neurons in the ganglia of the MP in the colon tissues of WT, *NLRP3* KO and *CASPASE-1/11* KO mice. 10 ganglia in the MP per mouse, n=6; DAPI, blue; Scale bar=25μM. ONE-way. ANOVA Bonferroni's Multiple Comparison Test; *P < 0.05, **P < 0.01, ***P < 0.001.

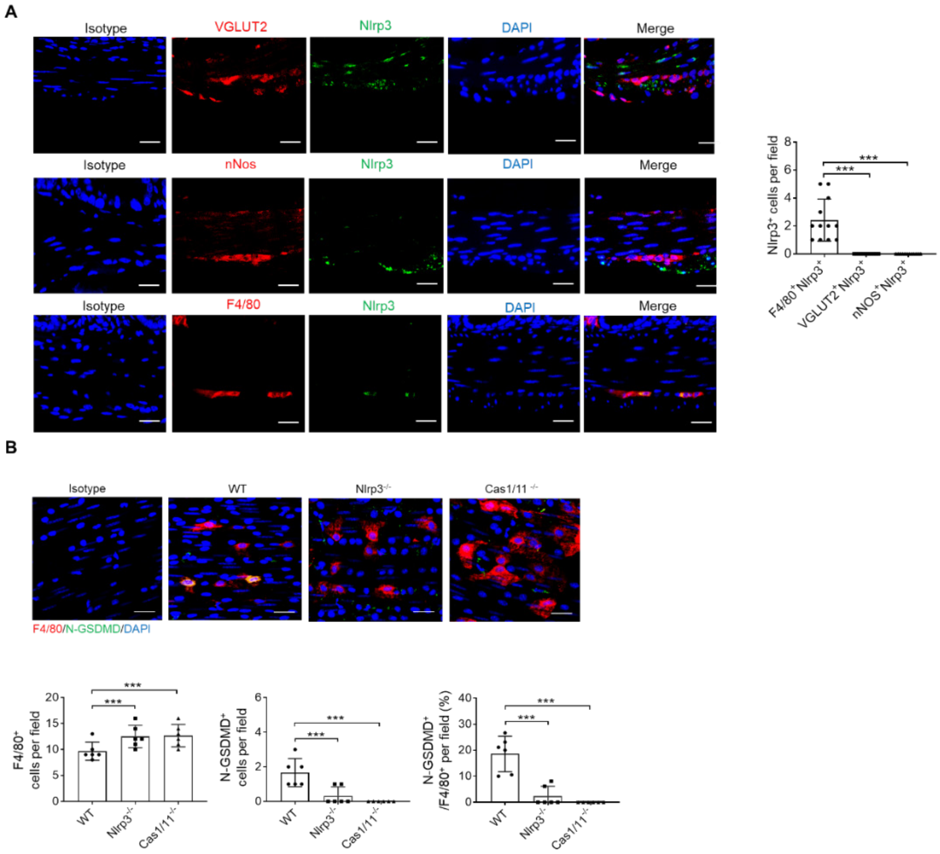

Figure S2. VGLUT2^{+} and nNOS^{+} neurons do not express NLRP3. (A) Colon neurons do not express NLRP3. Immunostaining of NLRP3 (Nlrp3) in F4/80+, HuC/D+, VGLUT2+ and nNOS+ cells in the ME of the colon sections from SPF mice. Histograms showed the percentage of NLRP3+ cells in F4/80+, HuC/D+, VGLUT2+ and nNOS+ cells. Isotype, antibody controls. 10 ganglia in the MP per mouse, n=6. Scale bar=40 μm; Blue, DAPI. (B) Immunostaining of N-GSDMD and F4/80 in the MP of the ME in the middle colon in SPF WT, NLRP3 KO and CASPASE-1/11 KO mice. 10 ganglia in the MP per mouse, n=6; DAPI, blue; Scale bar=25μM. ONE-way. ANOVA Bonferroni's Multiple Comparison Test; *P < 0.05, **P < 0.01, ***P < 0.001.

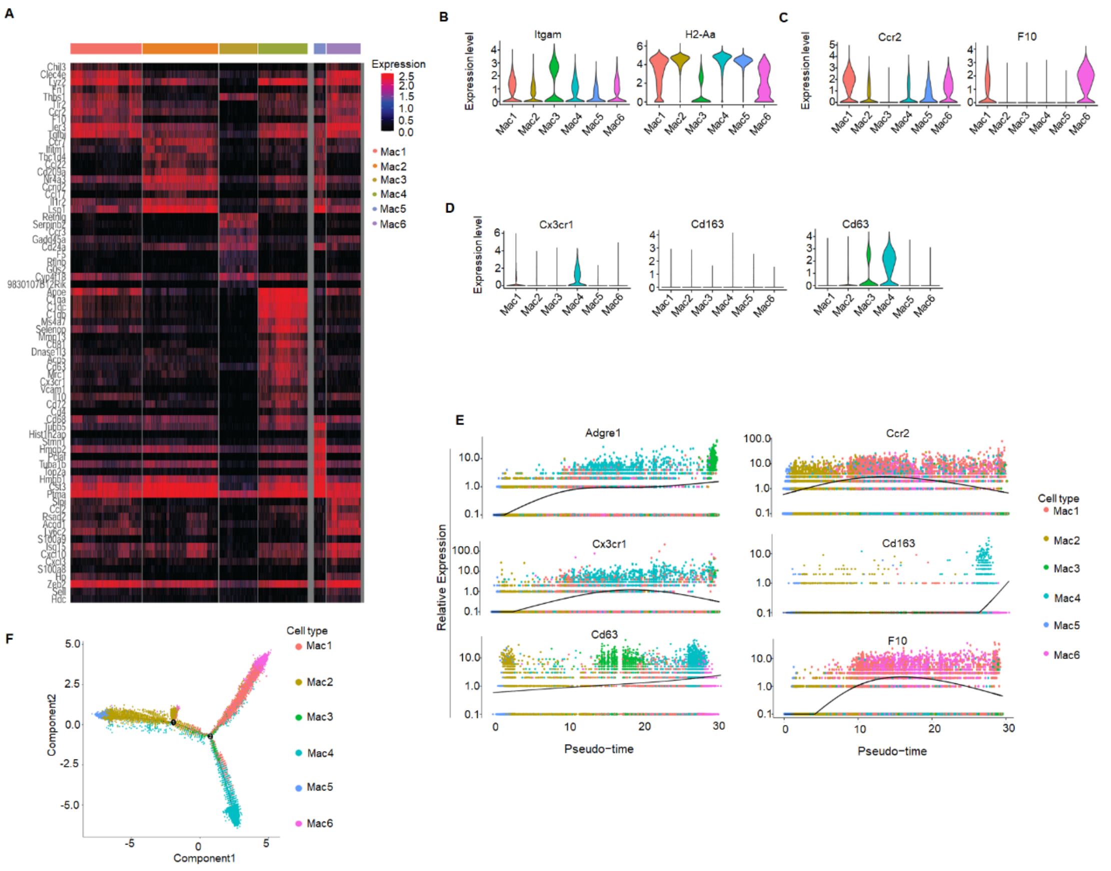

Figure S3. Trajectory analyses of selected enriched genes from the macrophage subsets during their development. (A) Heatmap reporting top differentially expressed genes of each cluster in Figure 3A. (B-D) Violin plots showing gene expression level of reported canonical marker genes of macrophage subpopulations across different clusters. (E) Trajectory analyses of selected enriched genes from macrophage subsets during their development, which covers the whole process from inflammatory macrophages to resident macrophages. (F) Developmental trajectory analysis of macrophage clusters with different cell clusters.

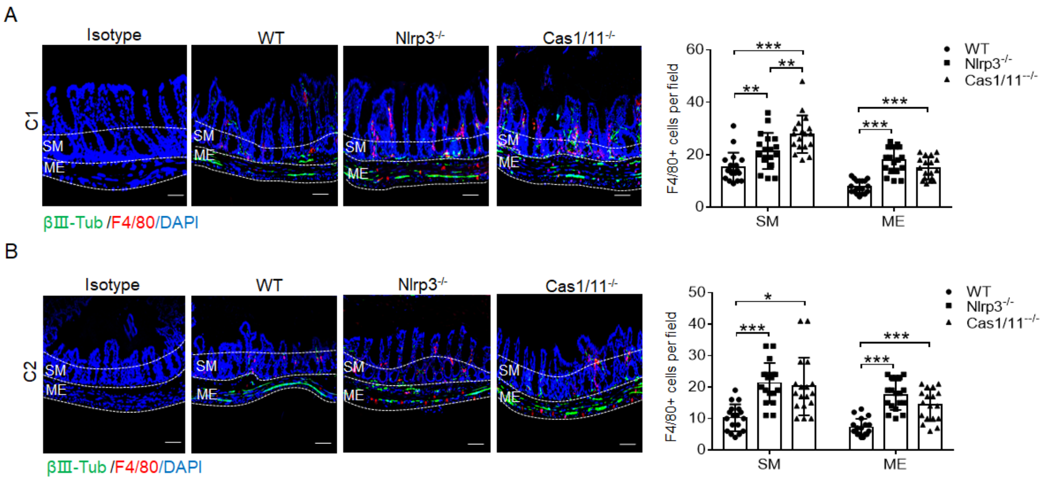

Figure S4. Immunostaining for βIII-Tub, F4/80 and DAPI in the submucosa and muscularis externa of small intestinal (A) or colon (B) sections from *CASPASE (CAS) 1/11 -/-*, *NLRP3 -/-* and WT mice. Small intestinal and colon segments stained with anti-βIII-Tubulin (green) or anti-F4/80 (red) antibodies and DAPI (blue). Histograms showed the number of βIII-Tub$^+$ and F4/80$^+$ cell in the submuscula (SM) and muscularis externa (ME) per field (100 ×). Scale bar=100 μm; 3 fileds (100×) per mouse, n=6. ONE-way ANOVA Bonferroni's Multiple Comparison Test; *P < 0.05, **P < 0.01, ***P < 0.001.

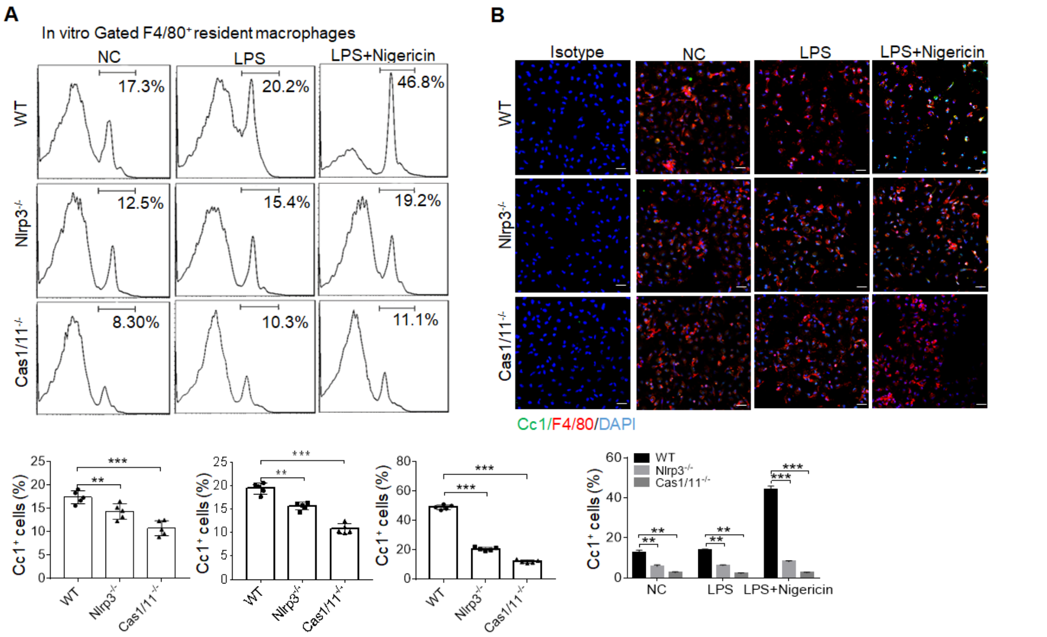

Figure S5. Analyses of resident macrophages from the colon tissues of mice. (A) Flow cytometry of Cc1$^+$cells in the gated F4/80$^+$ resident macrophages from isolated immune cells of the colon tissues of WT, *NLRP3 (Nlrp3) -/-* and *CASPASE (Cas) 1/11 -/-* mice. (B) Immunostaining of Cc1, F4/80 and DAPI in isolated cells from the colon

tissues of WT, *NLRP3 (Nlrp3) -/-* and *CASPASE (Cas)-1/11 -/-* mice. ONE-way ANOVA Bonferroni's Multiple Comparison Test; *P < 0.05, **P < 0.01, ***P < 0.001.

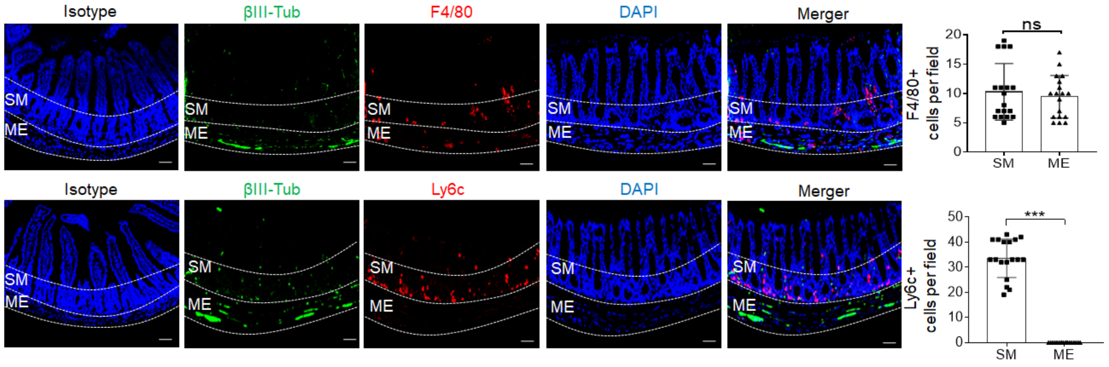

Figure S6. Distribution of F4/80^{+} and Ly6C^{+}macrophages in the colon tissues. Immunostaining of F4/80, Ly6c2, βIII-Tub and DAPI in whole mount colon sections from SPF mice. Histograms showed the number of F4/80^{+} and Ly6c2^{+} cells per field (100 ×) in the submucosa (SM) and muscularis externa (ME). Scale bar=100 μm; 3 fileds /mouse, n=6. Mann–Whitney U test; *P < 0.05, **P < 0.01, ***P < 0.001.

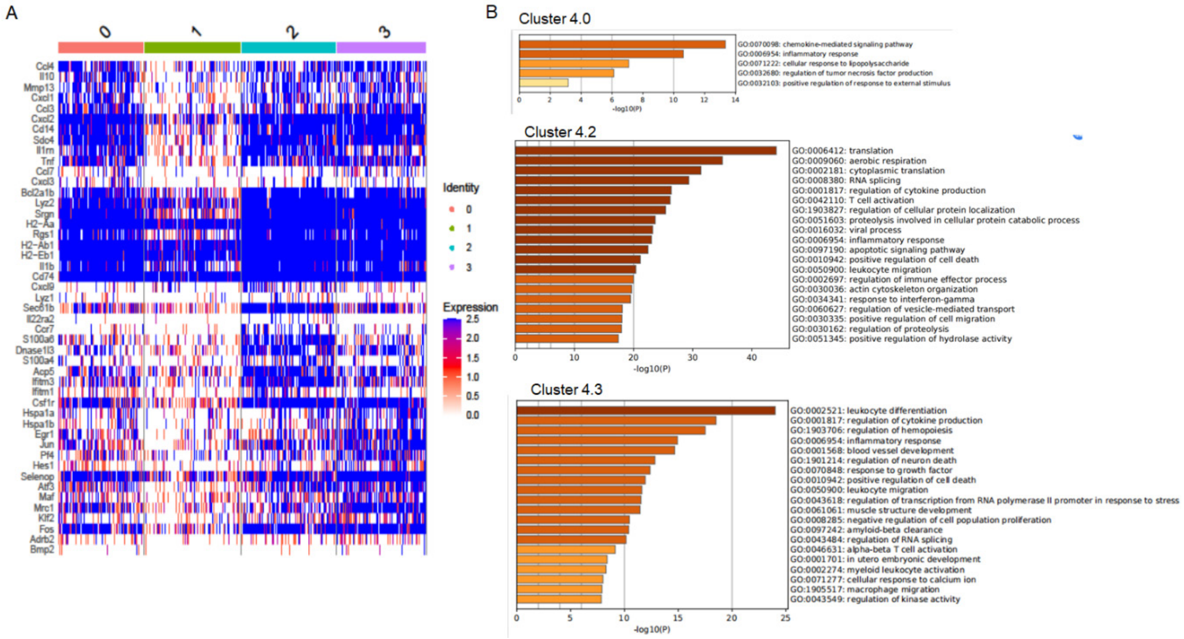

Figure S7. Heatmap reporting top differentially expressed genes in each subset of resident macrophages (A), and enrichment analyses of signal pathways in different subpopulations of resident macrophages (B).

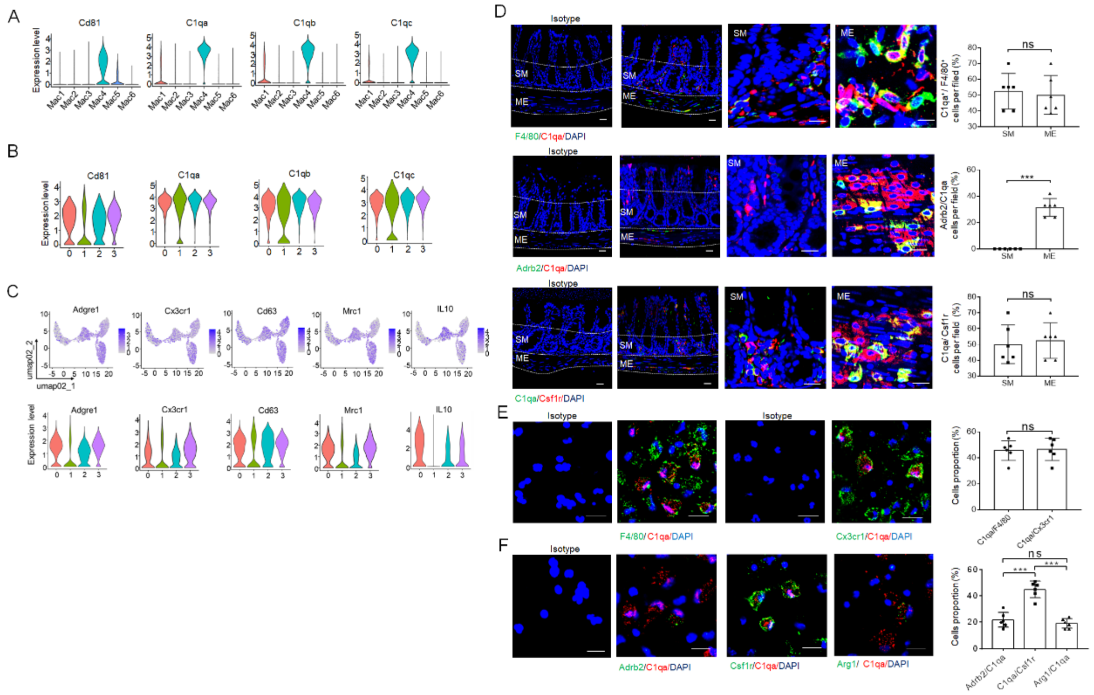

Figure S8. A subset of *c1qa*$^{+}$ cells exists in resident macrophage cluster. (A) Violin plots showing gene expression level of marker genes in resident macrophages across different clusters of macrophages. (B) Violin plots showing expression level of marker genes CD81, c1qa, c1qb and c1qc in different subpopulations of resident macrophages. (C) Feature plots (upper) and violin plots (lower) showing expression level of marker genes adgre1(F4/80), Cx3Cr1, CD63, Mrc-1(CD206) and IL-10 in different subpopulations of resident macrophages. (D) Immunostaining of c1qa/F4/80, adrb2/c1qa or c1qa/csf1r in submucosa and muscularis externa of colon sections. Scale bar=100 μM (SM+ME), 40 μM (SM) and 25 μM (ME); 10 ganglia in the myenteric plexus per mouse, n=6. DAPI, blue. (E) Immunostaining of c1qa/F4/80 and c1qa/CX3CR1 in isolated F4/80 macrophages by MicroBeads from the colon tissues of SPF mice. Scale bar=25 μM, n=6. (F) Immunostaining of adrb2/c1qa, c1qa/csf1r, and arg1/c1qa in isolated F4/80 macrophages by MicroBeads from the colon tissues of SPF mice. Scale bar=100 μM (SM+ME), 40 μM (SM) and 25 μM (ME), n=6; DAPI, blue; Mann–Whitney U test in C; Student's t-test in D and E, mean ±SD. $*P < 0.05$, $**P < 0.01$, $***P < 0.001$.

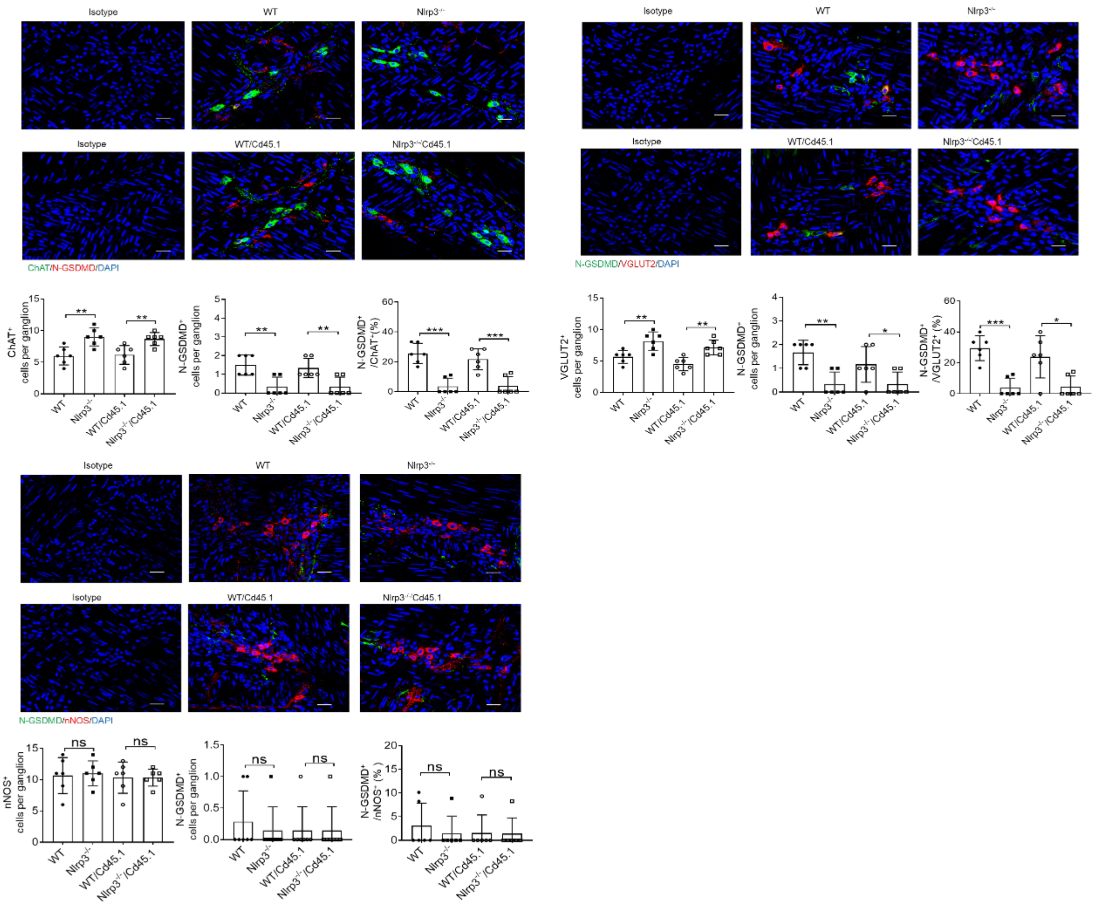

Figure S9. There are increased ChAT+ and VGLUT2+ neurons in NLRP3 KO /CD45.1 mice but not in WT/CD45.1 mice.N-GSDMD immunostaining of ChAT (N-GSDMD/ChAT), VGLUT2 (N-GSDMD/VGLUT2) and nNOS (N-GSDMD/nNOS) neurons in the MP of the colon of WT/CD45.1 and NLRP3 KO /CD45.1 mice. 10 ganglia in the MP per mouse, n=6 mice. Scale bar= 25µm. ONE-way ANOVA Bonferroni's Multiple Comparison Test; $*P < 0.05$, $**P < 0.01$, $***P < 0.001$.

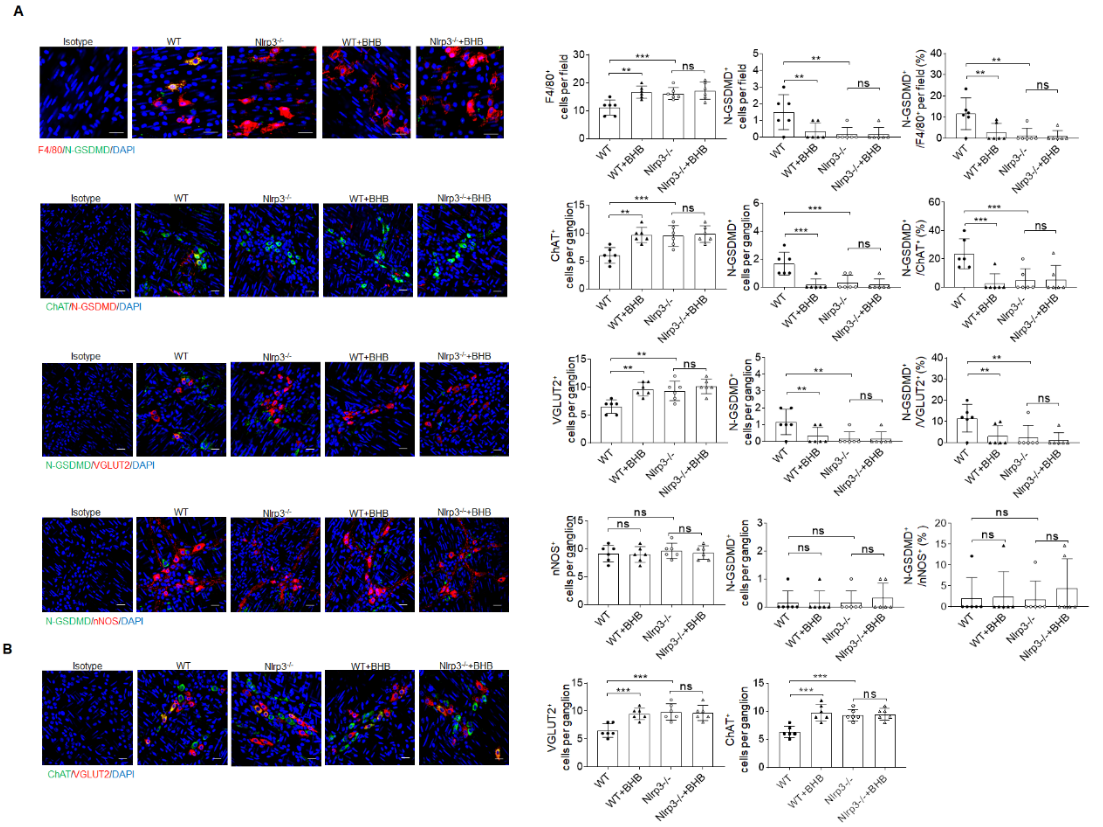

Figure S10. BHB treated mice have increased ChAT+ and VGLUT2+ neurons in WT mice but not NLRP3 KO mice. (A) N-GSDMD immunostaining of F4/80 (N-GSDMD/F4/80) macrophages, and ChAT (N-GSDMD/ChAT), VGLUT2 (N-GSDMD/VGLUT2) and nNOS (N-GSDMD/nNOS) neurons in the MP of BHB treated NLRP3 KO and WT mice. 10 ganglia in the MP per mouse, n=6 mice. Scale bar=25 µM. DAPI, blue; (B) Immunostaining for ChAT and VGLUT2 neurons in the ganglia of the MP of BHB treated NLRP3 KO and WT mice. 10 ganglia in the MP per mouse, n=6 mice. Scale bar=25 µM. DAPI, blue. ONE-way ANOVA Bonferroni's Multiple Comparison Test; *P < 0.05, **P < 0.01, ***P < 0.001.

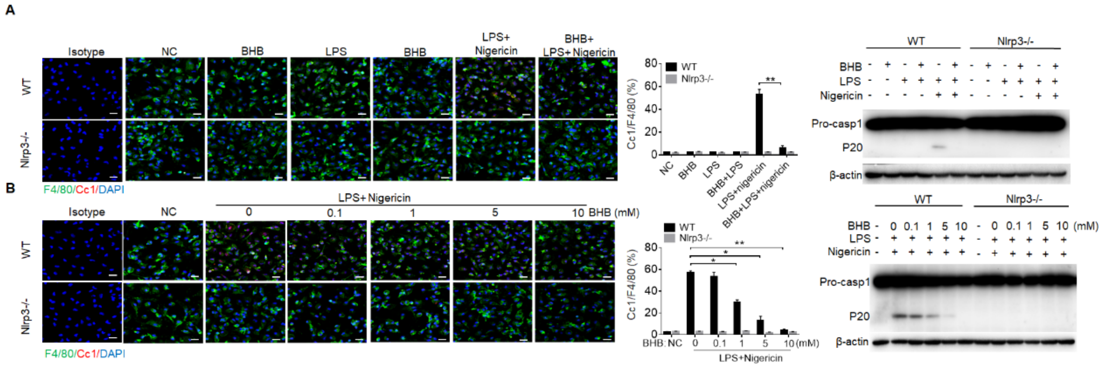

Figure S11. BHB treated gut resident macrophages resist against pyroptosis. (A) Immunostaining for Cc1/F4/80 in BHB treated gut macrophages and immunoblotting

of CASPASE 1 in the lysates of gut macrophages. Gut resident macrophages were isolated using anti-F4/80 MicroBeads. Scale bar=25μM. DAPI, blue. (B) Immunostaining of Cc1 and F4/80 in different concentrations of BHB treated gut macrophages and immunoblotting of CASPASE1 in the lysates of macrophages. Gut macrophages were isolated using anti-F4/80 MicroBeads. Scale bar=25μM. ONE-way ANOVA Bonferroni's Multiple Comparison Test. *P < 0.05, **P < 0.01, ***P < 0.001.

Table S1. Reagents and oligoes used in this paper.

REAGENT or RESOURCE	SOURCE	IDENTIFIER
**Antibodies**		
Percp-Cd11b M1/70 antibody	Biolegend	Cat: 101228
APC-CD45 30-F11 antibody	Biolegend	Cat: 103112
Percp-CD45 30-F11 antibody	Biolegend	Cat: 103132
PE-Ly6C antibody	Elabscience	Cat: E-AB-F1121D
APC-F4/80 BM8 antibody	Biolegend	Cat: 123116
PE-CD81 Eat2 antibody	BD Bioscience	Cat: 559519
APC-MHCII antibody	Elabscience	Cat: E-AB-F0990E
APC-CD81 Eat2 antibody	Biolegend	Cat: 104909
FITC-F4/80 BM8 antibody	Biolegend	Cat: 123108
Ly-6c (G-3) antibody	Santa	Cat: 10220
IL-10 (A-2) antibody	Santa	Cat: K0320
F4/80 (C-7) antibody	Santa	Cat: L3020
F4/80 antibody	RD	Cat: MAB5580
C1q-A (7HB) antibody	Santa	Cat: L1417
CD81 (B-11) antibody	Santa	Cat: L1820
βIII Tubulin (2G10) antibody	Abcam	Cat: Ab78078
Adrb2 antibody	Bioesn	Cat: BES2ER1
anti-CC1 antibody	Cell Signaling Technology	Cat: Asp296
Huc/d antibody	Abcam	Cat: Ab184267
Arg-1 antibody	Cell Signaling Technology	Cat: DEM3M
nNos antibody	ITI Biochem	Cat: ITI026657
Nlrp3 antibody	Affinity	Cat: DF7438
VGLUT2(8G9.2) antibody	Abcam	Cat: AB79157

N-GSDMD antibody	Affinity	Cat: DF13758
ChAT antibody	Protein tech	Cat: 20747-1-AP
βIII Tubulin antibody	Abcam	Cat: AB18207
β-Actin antibody	Protein tech	Cat: 81115-1-RR
Caspase1/p20/p10	Protein tech	Cat: 22915-1-AP
Cleaved IL-1 beta Antibody	Invitrogen	Cat: PA5-105048
N-GSDMD antibody	Abcam	Cat: ab215203
**Chemicals**		
Clodronate liposomes	Encapsula Nano Science	Cat: CLD8909-08302021
(R)-3-Hydroxybutanoic acid sodium	Sigma	Cat: 13613-65-5
Nigericin sodium	Selleckchem	Cat: S6653
Lipopolysaccharides	Sigma	Cat: 12181202
DMEM	Gibco	Cat: 11965118
Collagenase IV	Sigma	Cat: C5138
FBS	Gibco	Cat: 10099141
Dnase I	Solarbio	Cat: D8017
Percoll	Solarbio	Cat: P8370
